# Supplementary material for: Promiscuous interactions and protein disaggregases determine the material state of stress-inducible RNP granules
Source: eLife. 2015 Aug 4;4:e06807. doi: 10.7554/eLife.06807 (PMC4522596; doi:10.7554/eLife.06807)
Supplement: Supplementary file 1. — List of yeast strains used in this study. DOI: http://dx.doi.org/10.7554/eLife.06807.057 [file elife06807s001.docx]

**Yeast strains**

Table 1: *Saccharomyces cerevisiae* strains used in this study

| Name | Genetic background | Source |
| --- | --- | --- |
| BY4741 [RNQ+] | Mata, his3Δ1, leu2Δ0, met15Δ0, ura3Δ0, [RNQ+] | ATCC |
| BY4741 [RNQ+] Rnq1PD-sfGFP(V206) | BY4741 [RNQ+], pAG415GPD-Rnq1PD-sfGFP (V206) | this study |
| BY4741 [RNQ+] Rnq1PD-mCherry | BY4741 [RNQ+], pAG415GPD-Rnq1LC-mCherry | this study |
| BY4741 (Prion) Lsm4-mCherry | BY4741 [RNQ+], pAG415GPD-Lsm4-mCherry | this study |
| BY4741 (Prion) Lsm4-Dendra2 | BY4741 [RNQ+], pAG415GPD-Lsm4-Dendra2 | this study |
| BY4741 (Prion) Nrp1-mCherry | BY4741 [RNQ+], pAG416ADH-Nrp1-mCherry | this study |
| BY4741 (Prion) Nrp1-Dendra2 | BY4741 [RNQ+], pAG415GPD-Nrp1-Dendra2 | this study |
| A4713 [RNQ+] Nrp1-Cerulean | Mata, leu2-3,112; his3-11,-15; trp1-1; ura3-1; ade1-14; can1-100; [RNQ+]; sup35::KanMX; pAG416ADH1-NRP1 PLD-SUP35C, NRP1-Cerulean-HygB | Alberti et al., 2009 |
| A4713 [rnq-] Nrp1-Cerulean | Mata, leu2-3,112; his3-11,-15; trp1-1; ura3-1; ade1-14; can1-100; [rnq-]; sup35::KanMX; pAG416ADH1-NRP1 PLD-SUP35C, NRP1-Cerulean-HygB | Alberti et al., 2009, 3x on GdnHCl |
| BY4741 [rnq-] | Mata, his3Δ1, leu2Δ0, met15Δ0, ura3Δ0, [rnq-] | ATCC |
| BY4741 [rnq-] Rnq1PD-sfGFP(V206) | BY4741 [rnq-], pAG415GPD-Rnq1PD-sfGFP (V206) | this study |
| BY4741 [rnq-] Rnq1PD-mCherry | BY4741 [rnq-], pAG415GPD-Rnq1PD-mCherry | this study |
| BY4741 (PB) Lsm4-mCherry | BY4741 [rnq-], pAG415GPD-Lsm4-mCherry | this study |
| BY4741 (PB) Lsm4-Dendra2 | BY4741 [rnq-], pAG415GPD-Lsm4-Dendra2 | this study |
| BY4741 (SG) Nrp1-mCherry | BY4741 [rnq-], pAG416ADH-Nrp1-mCherry | this study |
| BY4741 (SG) Nrp1-Dendra2 | BY4741 [rnq-], pAG415GPD-Nrp1-Dendra2 | this study |
| BY4741 (SG) Nrp1-EGFP | BY4741 [rnq-], pAG415ADH-Nrp1 -EGFP | this study |
| BY4741 (SG) Nrp1∆PLD-EGFP | BY4741 [rnq-], pAG415ADH-Nrp1∆PLD-EGFP | this study |
| BY4741 (SG) Nrp1PLD-EGFP | BY4741 [rnq-], pAG415ADH-Nrp1PLD-EGFP | this study |
| BY4741 ∆hsp104 | BY4741 [rnq-], hsp104::KanMX4 | Giaever et al., 2002, 3x on GdnHCl |
| BY4741 ∆hsp104 Rnq1PD-sfGFP | BY4741 ∆hsp104, pAG415GPD-Rnq1PD-sfGFP (V206) | this study |
| BY4741 [RNQ+] Lsm4-GFP | BY4741 [RNQ+], Lsm4-GFP-His3MX | Huh et al., 2003 |
| BY4741 Lsm4-GFP (Prion) | BY4741 [RNQ+] Lsm4-GFP, pAG416GPD-Lsm4 | this study |
| BY4741 Lsm4-GFP (Prion), Dcp2-mCherry | BY4741 Lsm4-GFP (Prion), pAG415ADH-Dcp2-mCherry | this study |
| BY4741 Lsm4-GFP (Prion), Dcp1-mCherry | BY4741 Lsm4-GFP (Prion), pAG415ADH-Dcp1-mCherry | this study |
| BY4741 Lsm4-GFP (Prion), Edc3-mCherry | BY4741 Lsm4-GFP (Prion), pAG415ADH-Edc3-mCherry | this study |
| BY4741 Lsm4-GFP (PB) | BY4741 [rnq-], Lsm4-GFP-His3MX | Huh et al., 2003, 3x on GdnHCl |
| BY4741 Lsm4-GFP (PB), Dcp2-mCherry | BY4741 Lsm4-GFP (PB), pAG415ADH-Dcp2-mCherry | this study |
| BY4741 Lsm4-GFP (PB), Dcp1-mCherry | BY4741 Lsm4-GFP (PB), pAG415ADH-Dcp1-mCherry | this study |
| BY4741 Lsm4-GFP (PB), Edc3-mCherry | BY4741 Lsm4-GFP (PB), pAG415ADH-Edc3-mCherry | this study |
| BY4741 Dcp2-GFP | BY4741 [RNQ+], Dcp2-GFP-His3MX | Huh et al., 2003 |
| BY4741 Edc3-GFP | BY4741 [RNQ+], Edc3-GFP-His3MX | Huh et al., 2003 |
| BY4741 Pab1-GFP | BY4741 [RNQ+], Pab1-GFP-His3MX | Huh et al., 2003 |
| BY4741 Pub1-GFP | BY4741 [RNQ+], Pub1-GFP-His3MX | Huh et al., 2003 |
| BY4741 Ngr1-GFP | BY4741 [RNQ+], Ngr1-GFP-His3MX | Huh et al., 2003 |
| BY4741 Pbp1-GFP | BY4741 [RNQ+], Pbp1-GFP-His3MX | Huh et al., 2003 |
| BY4741 [RNQ+] Nrp1-GFP | BY4741 [RNQ+], Nrp1-GFP-His3MX | Huh et al., 2003 |
| BY4741 Nrp1-GFP (Prion) | BY4741 [RNQ+] Nrp1-GFP, pAG416GPD-Nrp1LC-Flag | this study |
| BY4741 Nrp1-GFP (Prion), Pab1-mCherry | BY4741 Nrp1-GFP (Prion), pAG415GPD-Pab1-mCh | this study |
| BY4741 Nrp1-GFP (SG) | BY4741 [rnq-], Nrp1-GFP-His3MX | Huh et al., 2003, 3x on GdnHCl |
| BY4741 Nrp1-GFP (SG), Pab1-mCherry | BY4741 Nrp1-GFP (SG), pAG415GPD-Pab1-mCh | this study |
| W303 Ade+ | Matalpha, can1-100, his3-11,15, leu2-3,112, trp1-1, ura3-1 | ATCC |
| W303 Pbp1-sfGFP (V206R) | W303 Ade+, Pbp1-sfGFP(V206R)-HisMX | this study |
| BY4741 [rnq-], sfGFP-µNS | BY4741 [rnq-], pAG415ADH-sfGFP (V206R)- µNS | this study |
| W303 ADE+ Hsp104-tdimer2 | Matalpha can1-100, his3-11,15, leu2-3,112, trp1-1, ura3-1, Hsp104-tdimer2-KanMX. | Alberti-lab, MPI-CBG |
| W303 ADE+ Hsp104-tdimer2, sfGFP-µNS | W303 ADE+ Hsp104-tdimer2, pAG415ADH-sfGFP (V206R)- µNS | this study |
| W303 ADE+ Hsp104-tdimer2, luciferase-sfGFP-µNS | W303 ADE+ Hsp104-tdimer2, pAG415ADH-luciferase(DM)-sfGFP (V206R)-µNS | this study |
| W303 ADE+ Hsp104-tdimer2, Ubc9ts-sfGFP-µNS | W303 ADE+ Hsp104-tdimer2, pAG415ADH-Ubc9ts-sfGFP (V206R)-µNS | this study |
| W303 ADE+ Hsp104-tdimer2, Nrp1-sfGFP-µNS | W303 ADE+ Hsp104-tdimer2, pAG415ADH-Nrp1-sfGFP (V206R)-µNS | this study |
| W303 ADE+ Hsp104-tdimer2, Nrp1∆PLD-sfGFP-µNS | W303 ADE+ Hsp104-tdimer2, pAG415ADH-Nrp1∆PLD-sfGFP (V206R)-µNS | this study |
| W303 ADE+ Hsp104-tdimer2, Nrp1PLD-sfGFP-µNS | W303 ADE+ Hsp104-tdimer2, pAG415ADH-Nrp1PLD-sfGFP (V206R)-µNS | this study |
| BY4741 [rnq-] Hsp104-GFP | Mata, his3Δ1, leu2Δ0, met15Δ0, ura3Δ0, [rnq-], HSP104-GFP-His3MX | Huh et al., 2003, 3x on GdnHCl |
| BY4741 [rnq-] Hsp104-GFP, Nrp1-mCherry | BY4741 [rnq-] Hsp104-GFP, pAG416ADH-Nrp1-mCherry | this study |
| BY4741 [rnq-] Hsp104-GFP, Edc3-mCherry | BY4741 [rnq-] Hsp104-GFP, pAG415ADH-Edc3-mCherry | this study |
| BY4741 Ssa1-tdimer2 | \|  \| BY4741 [rnq-], Ssa1-tdimer2-KanMX. \| \| --- \| --- \| | Alberti-lab, MPI-CBG, 3x on GdnHCl |
| BY4741 Ssa1-tdimer2, sfGFP-µNS | BY4741 Ssa1-tdimer2, pAG415ADH-sfGFP (V206R)- µNS | this study |
| BY4741 Ssa1-tdimer2, luciferase-sfGFP-µNS | BY4741 Ssa1-tdimer2, pAG415ADH-luciferase(DM)-sfGFP (V206R)-µNS | this study |
| BY4741 Ssa1-tdimer2, Ubc9ts-sfGFP-µNS | BY4741 Ssa1-tdimer2, pAG415ADH-Ubc9ts-sfGFP (V206R)-µNS | this study |
| BY4741 [rnq-] Ssa1-GFP | Mata, his3Δ1, leu2Δ0, met15Δ0, ura3Δ0, [rnq-], SSA1-GFP-His3MX | Huh et al., 2003, 3x on GdnHCl |
| BY4741 [rnq-] Ssa1-GFP, Nrp1-mCherry | BY4741 [rnq-] Ssa1-GFP, pAG416ADH-Nrp1-mCherry | this study |
| BY4741 [rnq-] Ssa1-GFP, Edc3-mCherry | BY4741 [rnq-] Ssa1-GFP, pAG415ADH-Edc3-mCherry | this study |
| BY4741 [rnq-] Hsp42-GFP | Mata, his3Δ1, leu2Δ0, met15Δ0, ura3Δ0, [rnq-], HSP42-GFP-His3MX | Huh et al., 2003, 3x on GdnHCl |
| BY4741 [rnq-] Hsp42-GFP, Nrp1-mCherry | BY4741 [rnq-] Hsp42-GFP, pAG416ADH-Nrp1-mCherry | this study |
| BY4741 [rnq-] Hsp42-GFP, Edc3-mCherry | BY4741 [rnq-] Hsp42-GFP, pAG415ADH-Edc3-mCherry | this study |
| BY4741 (SG) Nrp1-mCherry, Nrp1-sfGFP-µNS | BY4741 (SG) Nrp1-mCherry, pAG415ADH-Nrp1-sfGFP (V206R)- µNS | this study |
| BY4741 (SG) Nrp1-mCherry, Nrp1PLD-sfGFP-µNS | BY4741 (SG) Nrp1-mCherry, pAG415ADH-Nrp1PLD-sfGFP (V206R)- µNS | this study |
| BY4741 (SG) Nrp1-mCherry, Nrp1∆PLD-sfGFP-µNS | BY4741 (SG) Nrp1-mCherry, pAG415ADH-Nrp1∆PLD-sfGFP (V206R)- µNS | this study |
| BY4741 ∆nrp1 | BY4741 [rnq-], nrp1::KanMX4 | Giaever et al., 2002, 3x on GdnHCl |
| BY4741 ∆nrp1, Pub1-mCherry | BY4741 ∆*nrp1,* ADH-Pub1-mCherry | this study |
| BY4741 ∆nrp1 Pub1-mCherry, Nrp1-sfGFP-µNS | BY4741 ∆*nrp1* Pub1-mCherry, pAG415ADH-Nrp1-sfGFP (V206R)- µNS, | this study |
| BY4741 ∆nrp1 Pub1-mCherry, Nrp1PLD-sfGFP-µNS | BY4741 ∆*nrp1* Pub1-mCherry, pAG415ADH-Nrp1PLD-sfGFP (V206R)- µNS | this study |
| BY4741 ∆nrp1 Pub1-mCherry, Nrp1∆PLD-sfGFP-µNS | BY4741 ∆*nrp1* Pub1-mCherry, pAG415ADH-Nrp1∆PLD-sfGFP (V206R)- µNS | this study |
| BY4741 Pub1-mCherry | BY4741 [rnq-], Pub1-mCherry- HygB | this study |
| BY 4741 Pab1-mCherry | BY4741 [rnq-], Pab1-mCherry- HygB | this study |
| BY4741 Pub1-mCherry, sfGFPµNS | BY4741 Pub1-mCherry, pAG415ADH-sfGFP (V206R)- µNS | this study |
| BY4741 Pab1-mCherry, sfGFPµNS | BY4741 Pab1-mCherry, pAG415ADH-sfGFP (V206R)- µNS | this study |
| BY4741 Pub1-mCherry, Nrp1-sfGFP-µNS | BY4741 Pub1-mCherry, pAG415ADH-Nrp1-sfGFP (V206R)- µNS | this study |
| BY4741 Pub1-mCherry, Nrp1PLD-sfGFP-µNS | BY4741 Pub1-mCherry, pAG415ADH-Nrp1PLD-sfGFP (V206R)- µNS | this study |
| BY4741 Pub1-mCherry, Nrp1∆PLD-sfGFP-µNS | BY4741 Pub1-mCherry, pAG415ADH-Nrp1∆PLD-sfGFP (V206R)- µNS | this study |
| BY4741 Pub1-mCherry, luciferase-sfGFP-µNS | BY4741 Pub1-mCherry, pAG415ADH-luciferase(DM)-sfGFP (V206R)-µNS | this study |
| BY4741 Pab1-mCherry, luciferase-sfGFP-µNS | BY4741 Pab1-mCherry, pAG415ADH-luciferase(DM)-sfGFP (V206R)-µNS | this study |
| BY4741 [rnq-] Nrp1-mCherry, luciferase-sfGFP-µNS | BY4741 [rnq-], pAG416ADH-Nrp1-mCherry, pAG415ADH-luciferase(DM)-sfGFP (V206R)-µNS | this study |
| BY4741 [rnq-] luciferase-sfGFP-µNS, Nrp1∆PLD-mCherry | BY4741 [rnq-], pAG415ADH-luciferase(DM)-sfGFP (V206R)-µNS, pAG416ADH-Nrp1∆PLD-mCherry | this study |
| BY4741 [rnq-] luciferase-sfGFP-µNS, Nrp1PLD-mCherry | BY4741 [rnq-], pAG415ADH-luciferase(DM)-sfGFP (V206R)-µNS, pAG416ADH-Nrp1PLD-mCherry | this study |
| BY4741 [rnq-] luciferase-sfGFP-µNS, Hsp104-mCherry | BY4741 [rnq-], pAG415ADH-luciferase(DM)-sfGFP (V206R)-µNS, pAG416ADH-Hsp104-mCherry | this study |
| W303 Ade+ Nrp1-sfGFP(V206R) | W303 Ade+, Nrp1-sfGFP(V206R)-HisMX | this study |
| W303 Ade+ Lsm4-sfGFP(V206R) | W303 Ade+, Lsm4-sfGFP(V206R)-HisMX | this study |
| W303 Ade+ Nrp1-sfGFP(V206R), Edc3-mCherry | W303 Ade+, Nrp1-sfGFP(V206R), Edc3-mCherry-HygB | this study |
| W303 Ade+ ∆hsp104 | Matalpha, can1-100, his3-11,15, leu2-3,112, trp1-1, ura3-1, hsp104::KanMX | Alberti-lab, MPI-CBG |
| W303 Ade+ ∆hsp104 Nrp1-sfGFP(V206R) | W303 Ade+ ∆hsp104, Nrp1-sfGFP(V206R)-HisMX, | this study |
| W303 Ade+ ∆hsp104 Lsm4-sfGFP(V206R) | W303 Ade+ ∆hsp104, Lsm4-sfGFP(V206R)-HisMX, | this study |
| W303 Ade+ ∆sse1 | Matalpha, can1-100, his3-11,15, leu2-3,112, trp1-1, ura3-1, sse1::KanMX | Alberti-lab, MPI-CBG |
| W303 Ade+ ∆sse1 Nrp1-sfGFP(V206R) | W303 Ade+ ∆sse1, Nrp1-sfGFP(V206R)-HisMX | this study |
| W303 Ade+ ∆sse1 Lsm4-sfGFP(V206R) | W303 Ade+ ∆sse1, Lsm4-sfGFP(V206R)-HisMX | this study |
| W303 Ade+ ∆sse2 | Matalpha, can1-100, his3-11,15, leu2-3,112, trp1-1, ura3-1, sse2::KanMX | Alberti-lab, MPI-CBG |
| W303 Ade+ ∆sse2 Nrp1-sfGFP(V206R) | W303 Ade+ ∆sse2, Nrp1-sfGFP(V206R)-HisMX | this study |
| W303 Ade+ ∆sse2 Lsm4-sfGFP(V206R) | W303 Ade+ ∆sse2, Lsm4-sfGFP(V206R)-HisMX | this study |
| W303 Ade+ Edc3-sfGFP(V206R) | W303 Ade+, Edc3-sfGFP(V206R)-HisMX | this study |
| W303 Ade+ Edc3-sfGFP(V206R), luciferase-mCherry | W303 Ade+ Edc3-sfGFP (V206R), pAG415ADH-luciferase(DM)-mCherry | this study |
| W303 Ade+ Edc3-sfGFP(V206R), Ubc9ts-mCherry | W303 Ade+ Edc3-sfGFP (V206R), pAG415ADH-Ubc9ts-mCherry | this study |
| W303 Ade+ ∆hsp104 Edc3-sfGFP(V206R) | W303 Ade+ ∆hsp104, Edc3-sfGFP(V206R)-HisMX | this study |
| W303 Ade+ ∆sse1 Edc3-sfGFP(V206R) | W303 Ade+ ∆sse1, Edc3-sfGFP(V206R)-HisMX | this study |
| W303 Ade+ ∆sse2 Edc3-sfGFP(V206R) | W303 Ade+ ∆sse2, Edc3-sfGFP(V206R)-HisMX | this study |
| W303 Ade+ ∆hsp104 Edc3-sfGFP(V206R), luciferase-mCherry | W303 Ade+ ∆hsp104 Edc3-sfGFP(V206R), pAG415ADH-luciferase(DM)-mCherry | this study |
| W303 Ade+ ∆hsp104 Edc3-sfGFP(V206R), Pub1-mCherry | W303 Ade+ ∆hsp104 Edc3-sfGFP(V206R), Pub1-mCherry- HygB | this study |
| W303 Ade+ ∆hsp104 Edc3-sfGFP(V206R), luciferase-mCherry, GPD-Hsp104 | W303 Ade+ ∆hsp104 Edc3-sfGFP(V206R), luciferase-mCherry, pAG415GPD-Hsp104 | this study |
| W303 Ade+ ∆hsp104 Edc3-sfGFP(V206R), luciferase-mCherry, ADH-Hsp104 | W303 Ade+ ∆hsp104 Edc3-sfGFP(V206R), luciferase-mCherry, pAG415ADH-Hsp104 | this study |
| W303 Ade+ ∆hsp104 Edc3-sfGFP(V206R), luciferase-mCherry, SUP35-Hsp104 | W303 Ade+ ∆hsp104 Edc3-sfGFP(V206R), luciferase-mCherry, pAG416SUP35-Hsp104 | this study |
| BY4741 (SG) Nrp1-GFP, luciferase-mCherry | BY4741 Nrp1-GFP (SG), pAG415ADH-luciferase(DM)-mCherry | this study |
| BY4741 (SG) Nrp1-GFP, Ubc9ts-mCherry | BY4741 Nrp1-GFP (SG), pAG415ADH-Ubc9ts-mCherry | this study |
| W303 Ade+ Pbp1-sfGFP(V206R), luciferase-mCherry | W303 Pbp1-sfGFP (V206R), pAG415ADH-luciferase(DM)-mCherry | this study |
| W303 Ade+ Pbp1-sfGFP(V206R), Ubc9ts-mCherry | W303 Pbp1-sfGFP (V206R), pAG415ADH-Ubc9ts-mCherry | this study |
| W303 Ade+ Nrp1-sfGFP(V206R), luciferase-mCherry | W303 Ade+ Nrp1-sfGFP(V206R), pAG415ADH-luciferase(DM)-mCherry | this study |
| W303 Ade+ Nrp1-sfGFP(V206R), Ubc9ts-mCherry | W303 Ade+ Nrp1-sfGFP(V206R), pAG415ADH-Ubc9ts-mCherry | this study |
