# Supplementary material for: Promiscuous interactions and protein disaggregases determine the material state of stress-inducible RNP granules
Source: eLife. 2015 Aug 4;4:e06807. doi: 10.7554/eLife.06807 (PMC4522596; doi:10.7554/eLife.06807)
Supplement: Supplementary file 2. — Matlab routine for measuring the circularity of granules. DOI: http://dx.doi.org/10.7554/eLife.06807.058 [file elife06807s002.docx]

%% clearing command window and memory

clc;

clear;

close all;

%% Loading files and folders

read_folder='/Users/filepath';

cd(read_folder);

folder=dir('1*');

A=numel(folder);result=[];result_all=[];

for a=1:A

str1=folder(a).name;

str2=strcat(read_folder, '/',str1);

cd(str2);

files=dir('*.tif');

B=numel(files);

for b=1:B

img_ini=imread(files(b).name);

foci_mask = [];

foci_ID= 1; flag = 1;

while(flag)

foci_ID=1+foci_ID;

figure(1);title('Draw_roi');imagesc(img_ini);

h=imfreehand;

mask=uint16(h.createMask());

s=sum(sum(mask));

if (s > 10)

img=img_ini.*mask;

[row,col]=size(img);

img_ln=double(img(img>0));

img_mn=mean(img_ln);

img_sd=std(img_ln);

img_thresh = (img) > ((1*img_mn)+(0.5*img_sd));

cc = bwconncomp(img_thresh, 4);

labeled = labelmatrix(cc);

RGB_label = label2rgb(labeled, @spring, 'c', 'shuffle');

%figure(2), imshow(RGB_label);pause(0.2);

imgdata = regionprops(cc, 'all');

img_areas = [imgdata.Area];

[mx_area, idx] = max(img_areas);

img_foci = false(size(img_thresh));

img_foci(cc.PixelIdxList{idx}) = true;

area=imgdata(idx).FilledArea;

%figure(2), imshow(img_foci);pause(0.2);

img_edge = edge(img_foci, 'canny');

perimeter=sum(sum(img_edge));

merge = cat(3,(50*img_ini),imadjust(65535*uint16(img_edge)),zeros(row,col));

%figure(2);imshow(merge);pause(0.1);

circularity=(area*(4*pi))/(perimeter * perimeter)

eccen=imgdata(idx).Eccentricity;

result=cat(2,area, eccen, circularity);

result_all=cat(1,result_all,result)

else

flag=0;

end

end

end

end

cd(read_folder);

dlmwrite('Reasult_circularity_SG.txt',result_all)
